# Supplementary material for: Mass Spectrometry Detection and Imaging of a Non‐Covalent Protein–Drug Complex in Tissue from Orally Dosed Rats
Source: Angew Chem Int Ed Engl. 2022 Jul 13;61(36):e202202075. doi: 10.1002/anie.202202075 (PMC9542108; doi:10.1002/anie.202202075)
Supplement: Supplementary file 1 — Supporting Information [file ANIE-61-0-s001.pdf]

Angewandte Chemie
Eine Zeitschrift der Gesellschaft Deutscher Chemiker

Supporting Information

Mass Spectrometry Detection and Imaging of a Non-Covalent Protein–Drug Complex in Tissue from Orally Dosed Rats

*E. Illes-Toth, O. J. Hale, J. W. Hughes, N. Strittmatter, J. Rose, B. Clayton, R. Sargeant, S. Jones, A. Dannhorn, R. J. A. Goodwin, H. J. Cooper**

Supporting Information

Table of contents

Experimental section

Figure S1. Structure of bezafibrate.

Table S1. Study design.

Figure S2. Representative full scan native LESA mass spectra from thin liver tissue sections.

Figure S3. Native LESA CID MS/MS spectrum of FABP1.

Figure S4. Native LESA HCD MS/MS spectrum acyl-CoA binding protein.

Figure S5. Native LESA CID MS/MS spectrum of thymosin-β4.

Figure S6. Native LESA HCD MS/MS spectrum of ubiquitin 1-76.

Figure S7. Native LESA HCD MS/MS spectrum of somatic cytochrome c.

Figure S8. Native LESA mass spectra (low *m/z* region) from thin liver sections (2 hour timepoint).

Figure S9. Native LESA mass spectra (low *m/z* region) from thin liver sections (6 hour timepoint).

Figure S10. Replicate SIM-mode mass spectra obtained from three separate locations in liver sections from Animal 4 (2 hour timepoint) and Animal 6 (6 hour timepoint).

Figure S11. Nano-DESI PTCR mass spectrum of $[FABP1+bezafibrate+8H^{+}]^{8+}$ ions.

Table S2. Details of precursor ions selected for tPTCR and PTCR parameters.

Figure S12. Nano-DESI tPTCR mass spectrum from MSI experiment.

Figure S13. Targeted proton transfer charge reduction (tPTCR) nano-DESI mass spectrometry imaging.

Experimental section

Animal dosing and preparation of tissue

All procedures were conducted in accordance with the United Kingdom Animal (Scientific Procedures) Act 1986, approved by institutional ethical review committee (Babraham Institute Animal Welfare and Ethical Review Board) and conducted under Project Licence authority (PP7736793). All animal facilities have been approved by the United Kingdom Home Office Licensing Authority and meet all current regulations and standards of the United Kingdom.

Six male Han-Wistar rats (weight range 200-220g) were obtained from Charles River Laboratories (Margate, Kent, UK). Vendor-supplied health reports indicated that the rats were free of known viral, bacterial and parasitic pathogens. They were housed in groups of 3 in Tecniplast IVC cages (model number GR1800DD), in a 12 h light: dark cycle with room temperature maintained at 21°C +/- 2°C and relative humidity maintained at 55% +/- 10%, in a semi-barrier facility. The cages contained a 1–1.5 cm layer of 4 mm^3 Aspen chip bedding together with environmental enrichment (nestlet nesting material, Datesand) medium Aspen brick chew sticks (Datesand) and a cardboard play tunnel (Datesand). The rats had access to food (RM1 (E) IRR 0.25 pelleted diet, Special Diet Services, UK) and water ad libitum. Rats were allocated to cages on arrival and remained in the same social group throughout the study. Rats were test-naïve prior to the studies. Animal welfare was assessed throughout by daily monitoring of appearance, behaviour and cage environment. Rats were allowed to acclimatise to the animal unit for at least 1 week prior to in vivo work and were approximately 250g at experiment start.

Animal experiments and tissue dissection were performed by trained AstraZeneca staff. Rats were allocated to two groups: one group was euthanised 2 hours after dosing; the second group was euthanised 6 hours after dosing. In each group, two animals were dosed with the drug and one animal was vehicle-dosed (control). Bezafibrate was purchased from Sigma-Aldrich (Gillingham, UK) and a single oral dose of 150 mg/kg, (Vehicle 0.5% hydroxypropyl methycellulose (HPMC) and 0.1% Tween 80 in water) was administered via gavage. At 2 hours or 6 hours post-dose, the animals were

anaesthetised by administration of isofluorane, then euthanised by cutting the descending aorta and vena cava and death was confirmed by removal of the brain. Tissue samples were collected immediately and snap-frozen in isopentane over dry ice. Left lobes of livers were cut transversally, placed in ziplock bags to avoid deformations and stored at -80°C. Tissue sections were prepared 1-4 days before mass spectrometry analysis by cryosectioning (CM1810 Cryostat, Leica Microsystems, Wetzlar, Germany) at a thickness of 10 µM and thaw mounting onto glass slides. Sections were labelled, wrapped in aluminium foil and stored at -80°C until analysis. No washing procedures were performed to avoid any potential disruption of protein structure.

Liquid extraction surface analysis (LESA)

LESA sampling was performed by use of the Triversa Nanomate (Advion, Ithaca, NY, USA) platform coupled to either an Orbitrap Eclipse mass spectrometer or a modified research-only Q-Exactive HF mass spectrometer (both Thermo Fisher Scientific, Bremen, Germany). Images of the tissue sections were acquired using an Epson Perfection V300 flatbed photo scanner. The locations to be sampled were selected with the aid of LESA Points software (Advion). The LESA extraction solvent comprised 200 mM ammonium acetate (J.T. Baker, Deventer, The Netherlands) in in MS-grade water (Fisher Scientific, Loughborough, UK). "Contact" LESA sampling[1] was performed as follows: 1.5 µL of the solvent was aspirated from the solvent reservoir and dispensed onto the surface of the tissue for a dwell time of 60 s. Two µL was re-aspirated (to ensure all the sample was collected) and introduced to the mass spectrometer by chip-based nanoelectrospray. The Triversa NanoMate robot was operated with the ChipSoft 8.3.3 software at 1.65 kV capillary voltage and 0.35 psi pressure.

Nanospray desorption electrospray ionization (Nano-DESI)

Liver issue sections from Animal 3 were analysed by nano-DESI[2] on the Orbitrap Eclipse. A home-built nano-DESI ion source was constructed from an XYZ-stage (Zaber Technologies Inc., Vancouver, Canada) mounted at the mass spectrometer inlet. Fused silica capillaries were flame-pulled (O.D. 275 µm, I.D. 75 µm prior to modification) and cut to a final outer diameter of approx. 100 µm. The sampling capillary was positioned directly in front of the mass spectrometer inlet to aspirate solvent with the

inlet vacuum. The exit of the sampling capillary was not flame-pulled but had its coating removed. Solvent was delivered at a rate of 1.9 μL/min through the first capillary by a 10 mL gas-tight syringe (Hamilton, Reno, NV) and a liquid junction was formed between the two capillaries. The liquid junction covered a region of tissue approx. 200 μm in diameter. High voltage was supplied from the mass spectrometer to the syringe needle and optimized for ion intensity and spray stability (0.8 kV for MSI). The ion transfer tube was set to 250 °C.

Stage movement was controlled by Zaber Control (Zaber Technologies Inc.) or by custom software written in LabVIEW (NI, Austin, Texas). MSI was performed by positioning a tissue section underneath the nano-DESI probe and scanning the probe across the surface at 5 μm/s. Sequential lines were acquired with a line spacing of 200 μm (ion image generation from line scans is described below). A relay connected to an Arduino Uno v3 microcontroller triggered contact closure to signal the start of a new line scan in MSI experiments.

Mass spectrometry

Positive ion mode full scan mass spectra were collected by use of an Orbitrap Eclipse mass spectrometer (Thermo Fisher Scientific, Bremen, Germany) with settings as follows: 100% AGC target, 60 RF%, 2 microscans, 500 ms injection time, 120,000 resolution, ions were detected with the orbitrap in the range of *m/z* 300-3000 using protein mode, 250°C ion transfer tube temperature, standard pressure. All mass spectra were analysed in FreeStyle v. 1.4 (Thermo Fisher Scientific) and exported as csv files for subsequent plotting in GraphPad Prism v. 6.1 and Origin 2016.

SIM-mode mass spectra were also acquired by use of the Orbitrap Eclipse mass spectrometer with settings as follows: AGC target 100%, RF 60%, microscans 2, resolution 120,000, SIM scan mode, scan low *m/z* 1831.5, scan high *m/z* 1839.5, protein mode, standard pressure, 250°C ion transfer tube temperature. Mass spectra presented comprised 50 summed scans. Mass spectra were analysed in FreeStyle v. 1.4, exported as csv files and plotted in GraphPad Prism v. 6.1.

Tandem mass spectra for identification of proteins present in the liver were acquired by use of the Orbitrap Eclipse with settings as follows: AGC target 100%, RF% 60, microscans 2, injection time 500 ms unless otherwise stated, in-source fragmentation 30 - 60 V, resolution 120,000, applying HCD (normalised collision energy, NCE, 35-38%) or CID (NCE 36%) fragmentation following ion trap (for *m/z* > 2000) or quadrupole (for *m/z* < 2000) isolation of precursor ions with a 2-2.5 Da window, respectively. Fragment ions were detected in the orbitrap in the range of *m/z* 350-3000 or *m/z* 500-5000, using protein mode, standard pressure, 250°C ion transfer tube temperature. Uniprot proteome was downloaded as a non-compressed xml file for all proteins of *Rattus norvegicus* with reference UP000002494 and imported for analysis. Fragments were searched against this database and interrogated in ProSight[3] 4.1 and ProSight Lite[3], followed by manual verification in MS Product of Protein Prospector[4] v. 6.2.2. Settings for ProSight were: database 30 ppm tolerance, precursor search window 1000 Da, monoisotopic, minimum fragments 4, maximum number of proteins to return 25, S/N ratio 2, min RL value 0.9, fragment ion tolerance 15 ppm, maximum mass 60,000, max charge 25, first *m/z* 400, last *m/z* 4000, CID or HCD fragmentation, get top N peaks 5, and the *m/z* of monoisotopic precursors were manually entered for individual searches, all PTMs were considered and the Δm mode was on.

Tandem mass spectra for identification of the [FABP1-bezafibrate] complex were acquired by use of a modified research-only Thermo Orbitrap QE-HF, whereby customized instrument control software, provided in collaboration with Thermo Fisher Scientific, allowed for the application of the following settings: S-lens RF 40%, first mass 300 *m/z*, last mass 3000 *m/z*, isolation range low ~1835 *m/z*, isolation range high ~1836 *m/z*, max injection time 200 ms, resolution 7500, HCD energy (NCE) 7%, AGC target $1e^6$, averaging 2, noise threshold 3, source DC offset (V) 22 V, injection flatapole (V) 5.0 V, interflatapole lens (V) 4.0, bent flatapole DC (V) 2.0, transfer multipole DC (V) 1, diagnostic trapping 1, profile spectrum, C-trap charge detector on. Approximately 200-300 scans were collected and visualised in XCalibur 4.2 Qual Browser and Thermo Scientific FreeStyle v. 1.4, exported as csv files and plotted in GraphPad Prism v. 6.1.

Nano-DESI MSI was performed on the Orbitrap Eclipse mass spectrometer by multiplexed targeted proton transfer charge reduction (tPTCR) of intact proteins. S-lens RF was set to 40%, and source collision voltage was "off". Precursor ions for three proteins and the [FABP1-bezafibrate] complex (see *Table S2, Supporting Information*) were isolated sequentially for up to 250 ms using the quadrupole mass analyser and stored together in the ion routing multipole in N_2 bath gas set to a pressure of 8 mTorr. All isolated ions were then passed to the high-pressure cell of the linear ion trap and underwent PTCR for 7 ms with approx. $2x10^5$ perfluoroperhydrophenanthrene anions. Ions were then transmitted from the ion trap to the orbitrap analyser and detected over the range *m/z* 1650-3000 with the resolution set to 7500 (defined at *m/z* 200). Approximately one scan was recorded per second.

Ion images were generated by conversion of Thermo raw datafiles into a single imzML file using Firefly (v.3.2.0.23, Prosolia, Inc., Indianapolis, IN). Signals from approx. 24 scans were summed per pixel, resulting in a final pixel size of 200 x 200 μm. The imzML file was processed in MSiReader[5] with 1x linear interpolation and a linear intensity scale. Ion images for specific *m/z* (± *m/z* 0.1) were exported as MATLAB fig files. Ion images composed of multiple charge states of the same protein were produced by summing data from fig files using custom scripts in MATLAB (R2019a, MathWorks, Natick, USA).

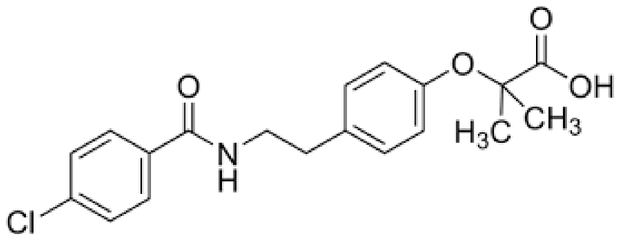

Figure S1. Structure of bezafibrate, $C_{19}H_{20}NO_4Cl$, molecular weight 361.1081 Da.

Animal number	Time point	Dose
**1**	2 h	Control (vehicle-dosed)
**2**	6 h	Control (vehicle-dosed)
**3**	2 h	Bezafibrate (150 mg/kg)
**4**	2 h	Bezafibrate (150 mg/kg)
**5**	6 h	Bezafibrate (150 mg/kg)
**6**	6 h	Bezafibrate (150 mg/kg)

Table S1. Study design.

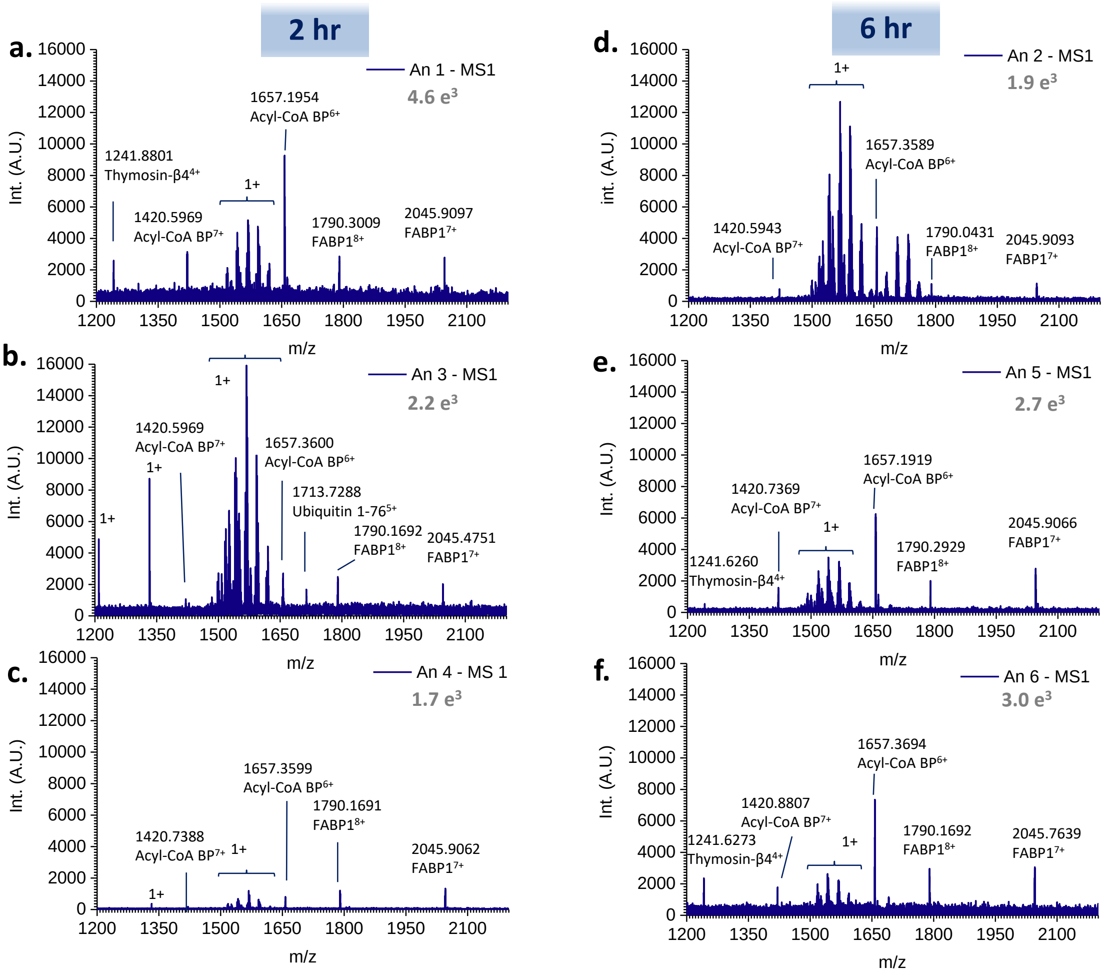

Figure S2. Representative full scan native LESA mass spectra from thin liver tissue sections. All sampling locations were towards the outer edge of the liver. Left column: 2 hours post-dose a) vehicle-dosed control; b) and c) bezafibrate-dosed biological replicates; Right column: 6 hours post-dose d) vehicle-dosed control; e) and f) bezafibrate-dosed biological replicates. The intensity of free FABP1 (7+ and 8+) ions are indicated in the top right of each mass spectrum in grey. Other proteins detected include acyl-CoA binding protein (Acyl-CoA BP), thymosin-β4 and ubiquitin 1-76 fragment. Singly-charged lipid ions are indicated with a bracket and 1+ notation.

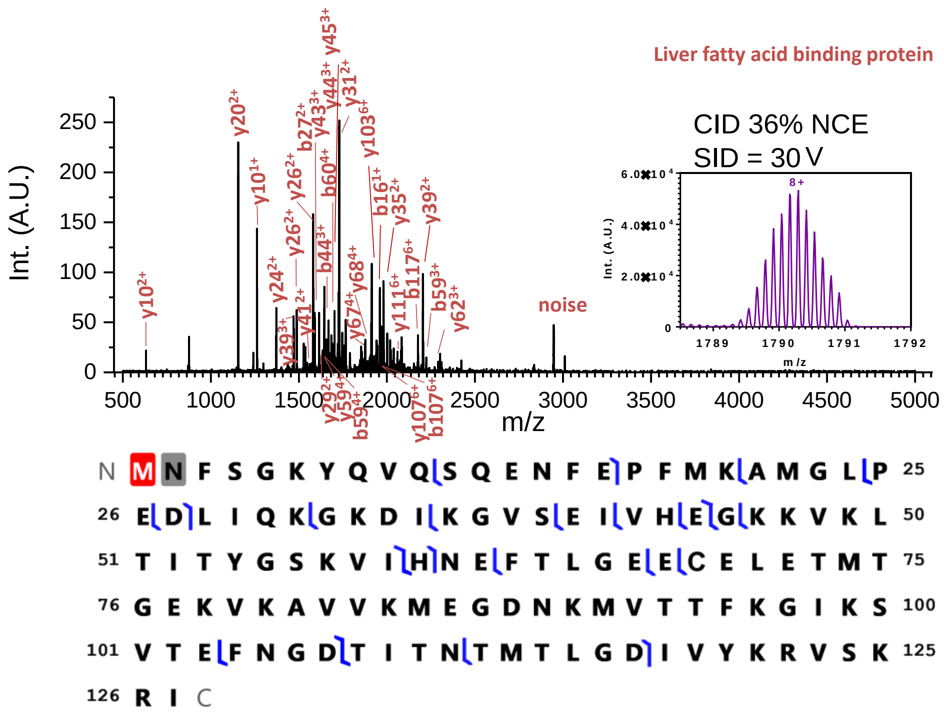

Figure S3. Native LESA CID MS/MS spectrum of FABP1 acquired at 36% NCE, 30 V in-source fragmentation (SID) following selection of the 8+ precursor ion (*m/z* 1790). The 8+ precursor ion is shown in purple (~20 scans), and the assigned b and y ions are shown in red in the tandem mass spectrum (~110 scans). The location of fragment ions are indicated in the amino acid sequence processed in ProSight[3] v. 4.1 and ProSight Lite[6], followed by manual checks in Protein Prospector v. 6.2.2., MS Fragment[4]. The mass of FABP1 was consistent with an N-terminal acetylation of Met (red square) and a deamidation of Asp (grey square), giving an observed monoisotopic mass of 14306.30 Da (Δm: 0.002 Da; 0.16 ppm as compared to the calculated value).

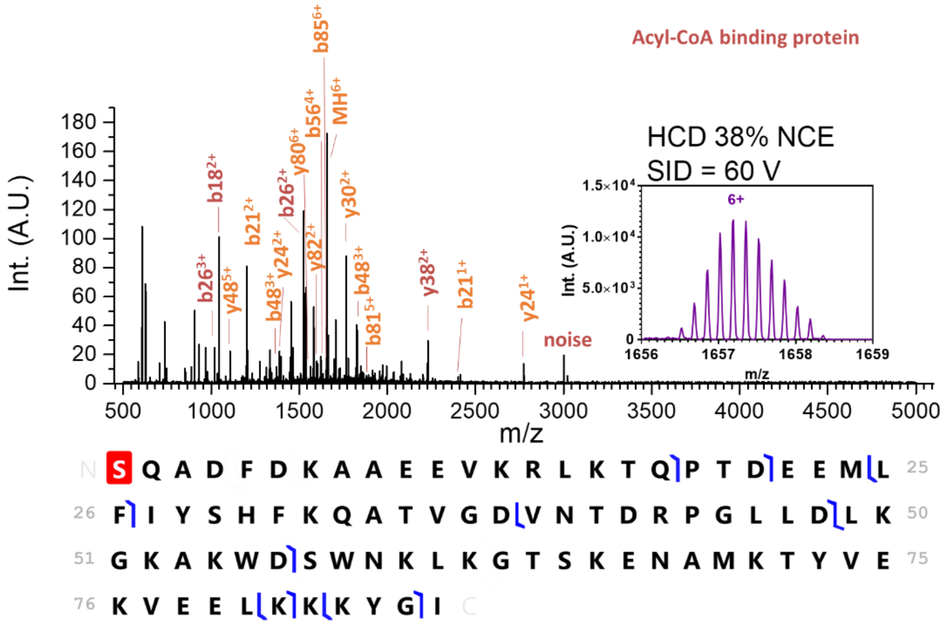

Figure S4. Native LESA HCD MS/MS spectrum of acylCoA binding protein obtained at 38% NCE, 60 V in-source fragmentation (SID) selecting the 6+ precursor ion (*m/z* 1657). The 6+ precursor ion is displayed in purple (~40 scans), and the assigned b and y ions are shown in red in the tandem mass spectrum (~150 scans). The location of fragment ions are indicated in the amino acid sequence processed in ProSight[3] v. 4.1 and ProSight Lite[6], followed by manual checks in Protein Prospector v. 6.2.2.,MS Fragment[4]. The experimentally determined mass of acyl-CoA binding protein was consistent with removal of the first Met residue and acetylation of Ser (red square), giving an observed monoisotopic mass of 9932.06 Da (Δm: -0.063 Da; -6.35 ppm deviation from the calculated value).

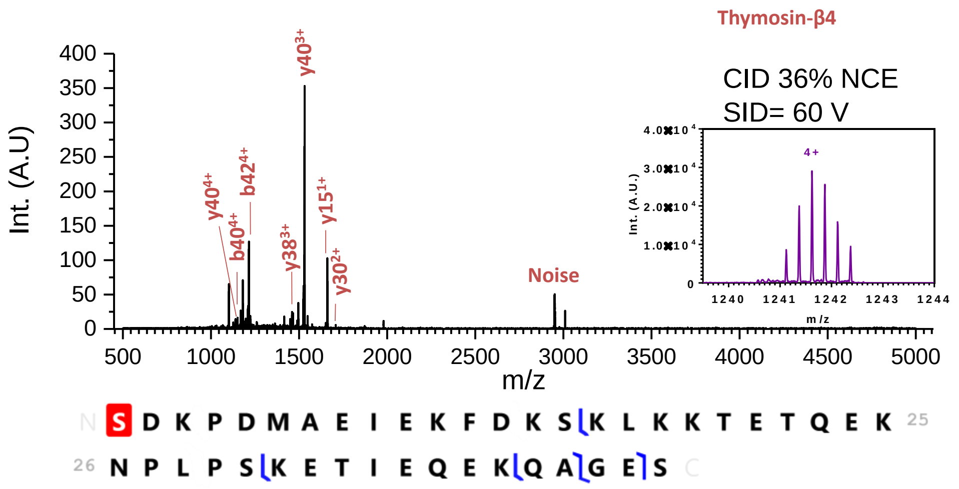

Figure S5. Native LESA CID MS/MS spectrum of thymosin-β4 acquired at 36% NCE, 60 V in-source fragmentation (SID) following selection of the 4+ precursor ion (*m/z* 1242). The 4+ precursor ion can be seen in purple (~30 scans), and the assignment of b and y ions are shown with red in the tandem mass spectrum (~140 scans). The location of fragment ions are illustrated in the amino acid sequence analysed in ProSight[3] v. 4.1 and ProSight Lite[6], followed by manual verification in Protein Prospector v. 6.2.2., MS Fragment[4]. The mass of thymosin-β4 revealed acetylation of Ser (red square) on the first residue, corresponding to an observed monoisotopic mass of 4960.47 Da (Δm: -0.013 Da; -2.61 ppm difference from the calculated value).

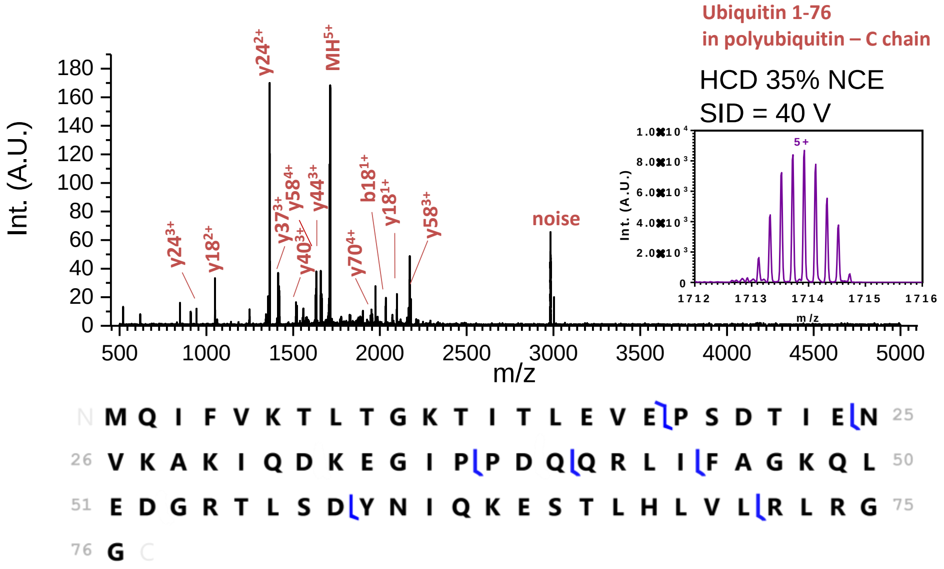

Figure S6. Native LESA HCD MS/MS spectrum of ubiquitin 1-76 attained at 35% NCE, 40 V in-source fragmentation (SID) of the 5+ precursor ion. The 5+ precursor ion is indicated in purple (~30 scans), and the resultant b and y ions can be seen in red in the tandem mass spectrum (~140 scans). The position of fragment ions are shown in the amino acid sequence analysed in ProSight[3] v. 4.1 and ProSight Lite[6], following verification in Protein Prospector v. 6.2.2., MS Fragment[4]. The experimentally determined mass of ubiquitin 1-76 gave an observed monoisotopic mass of 8859.60 Da (Δm: -0.012 Da; -1.40 ppm difference compared to the calculated value).

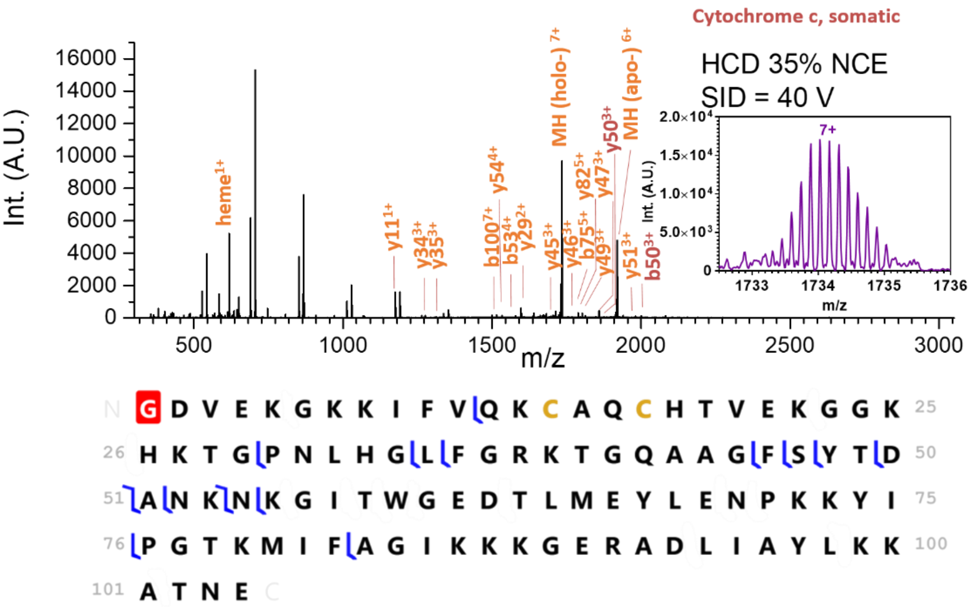

Figure S7. Native LESA HCD MS/MS spectrum of somatic cytochrome c obtained at 35% NCE, 40 V in-source fragmentation (SID) of the 7+ precursor ion. The 7+ precursor ion is indicated in purple (~10 scans), assignment of b and y ions are shown in red in the tandem mass spectrum (~60 scans). The position of fragment ions are indicated in the amino acid sequence analysed in ProSight[3] v. 4.1 and ProSight Lite[6], following manual verification in Protein Prospector v. 6.2.2., MS Fragment[4]. The experimentally determined mass of cytochrome c gave an observed monoisotopic mass of 12125.12 Da (Δm: -0.050 Da; -4.10 ppm difference compared to the calculated value), consistent with an acetylation of Gly (red square). Heme is covalently bound to the Cys residues[7] (yellow case) in cytochrome c as indicated by the 6+ holo-form of precursor ion. Heme is disrupted and released upon HCD fragmentation revealed by the charge reduced apo-precursor (5+) and the appearance of heme ion.

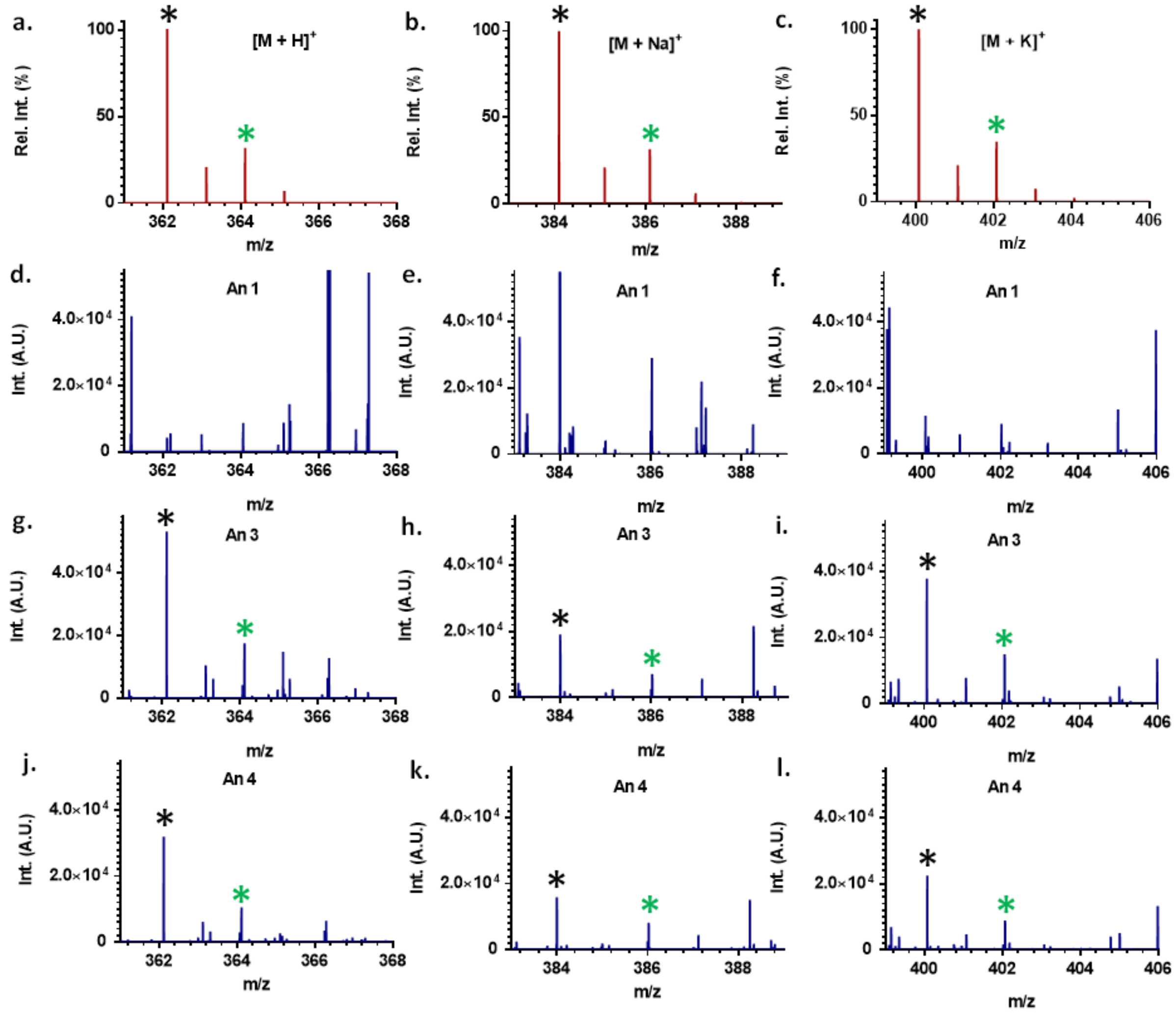

Figure S8. Native LESA mass spectra (low *m/z* region) from thin liver sections (2 hour timepoint). Comparison of simulated mass spectra for (a) protonated bezafibrate, (b) sodiated bezafibrate and (c) potassiated bezafibrate with experimental mass spectra obtained from (d)-(f) control tissue (animal 1) and (g)-(l) dosed tissue (animals 3 and 4). Simulated mass spectra were tabulated in and exported from isoPro 3.0 based on the Yergey[8] algorithm adapted by M. W. Senko. Experimental mass errors were between 4-5 ppm. * indicates monoisotopic peak, * indicates ^{37}Cl isotope peak

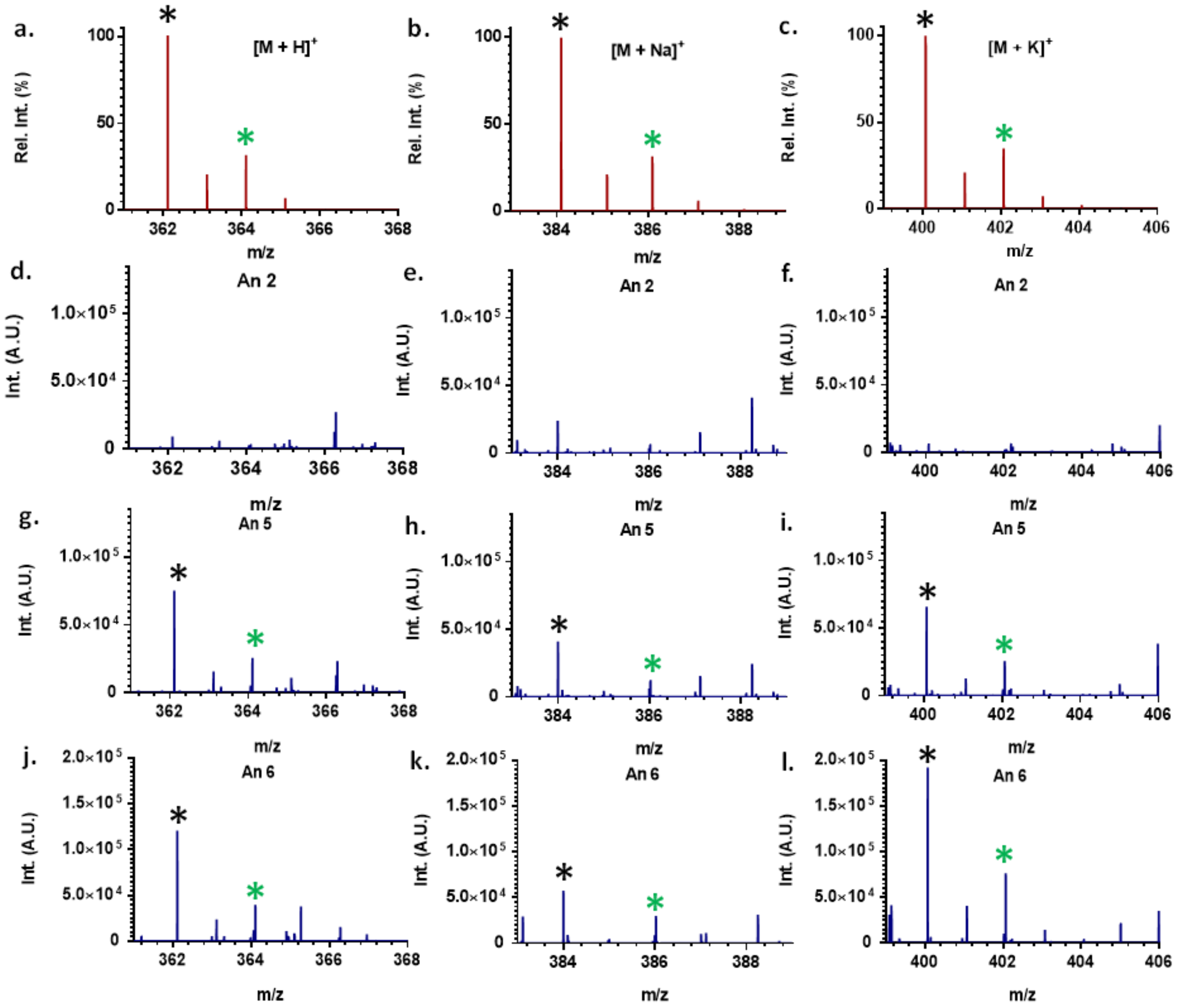

Figure S9. Native LESA mass spectra (low *m/z* region) from thin liver sections (6 hour timepoint). Comparison of simulated mass spectra for (a) protonated bezafibrate, (b) sodiated bezafibrate and (c) potassiated bezafibrate with experimental mass spectra obtained form (d)-(f) control tissue (animal 2) and (g)-(l) dosed tissue (animals 5 and 6). Simulated mass spectra were tabulated in and exported from isoPro 3.0 based on the Yergey[8] algorithm adapted by M. W. Senko. Experimental mass errors were between 3-5 ppm. * indicates monoisotopic peak, * indicates ^{37}Cl isotope peak.

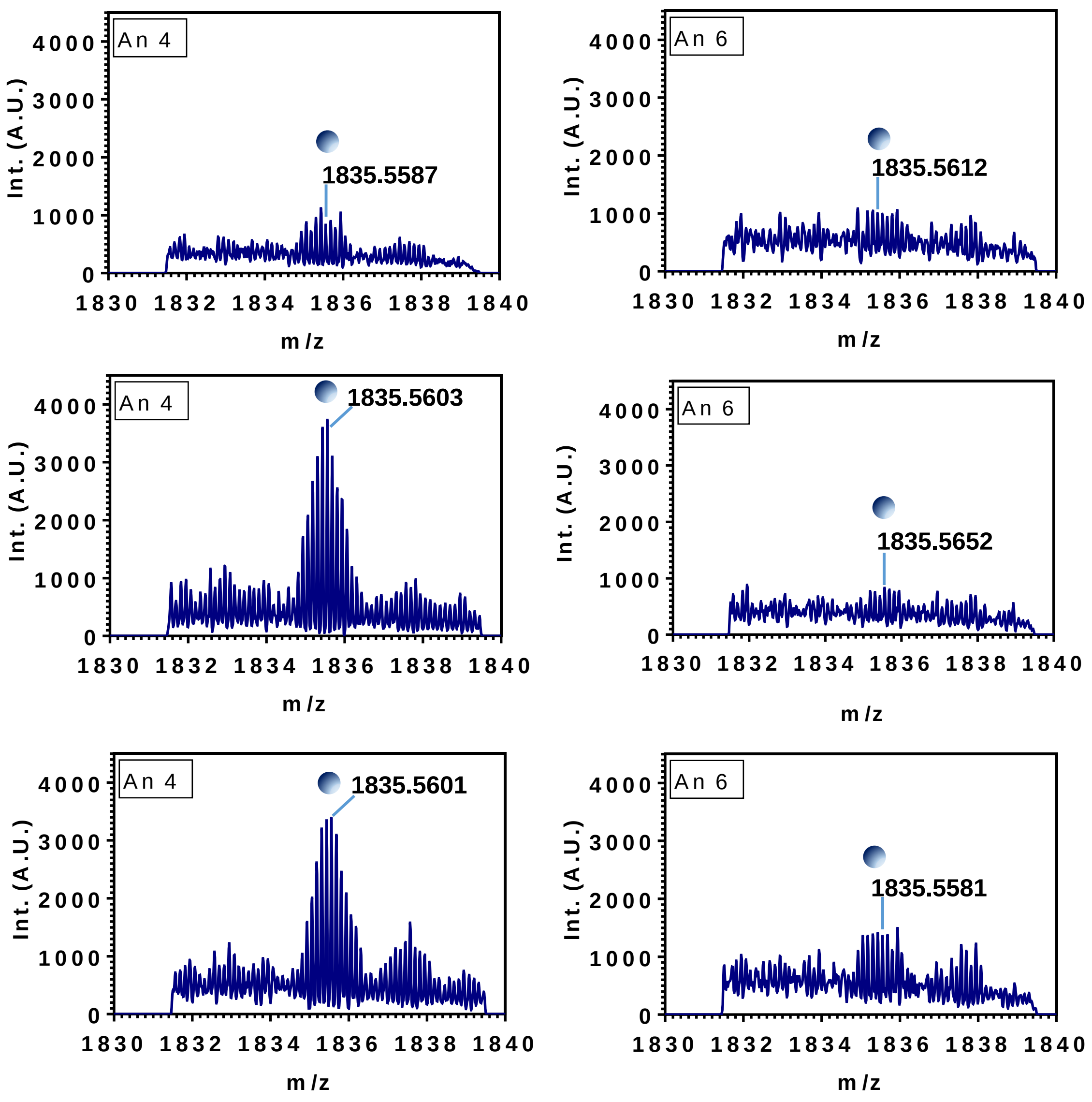

Figure S10. Replicate SIM-mode mass spectra obtained from three separate locations in liver sections from Animal 4 (2 hour timepoint) and Animal 6 (6 hour timepoint). m/z of most abundant isotope peak is given.

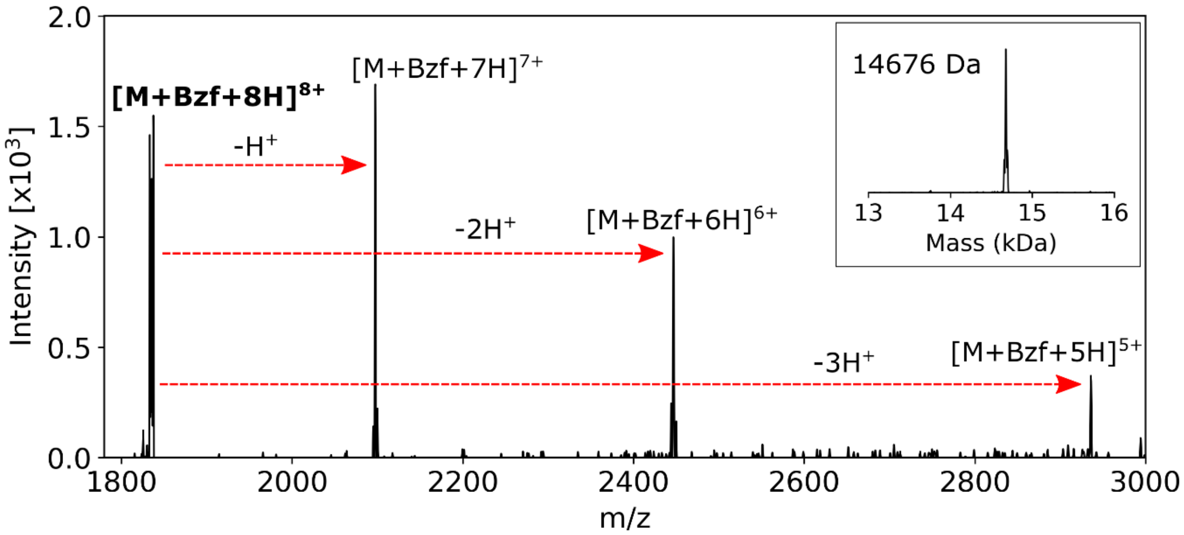

Figure S11: nano-DESI PTCR mass spectrum for [FABP+bezafibrate+8H$^+$]$^{8+}$ ions from liver tissue of Animal 3. The precursor ion signal is indicated in bold. The inset shows the deconvoluted experimental average mass = 14676 Da, theoretical mass = 14677 Da. Each proton transfer reaction reduced the charge state of the precursor ions by one.

	Precursor *m/z*	Precursor z	Isolation window width (*m/z*)	AGC target (charges)	Max. injection time (ms)	PTCR reaction time (ms)
FABP	1790	8	3	5x10^4	250	7
Acyl-coA BP	1657	6	3	5x10^4	250	7
a^H-globin	1977	8	3	5x10^4	250	7
FABP+Bzf	1835	8	3	5x10^4	250	7

Table S2: Details of precursor ions selected for tPTCR and PTCR parameters.

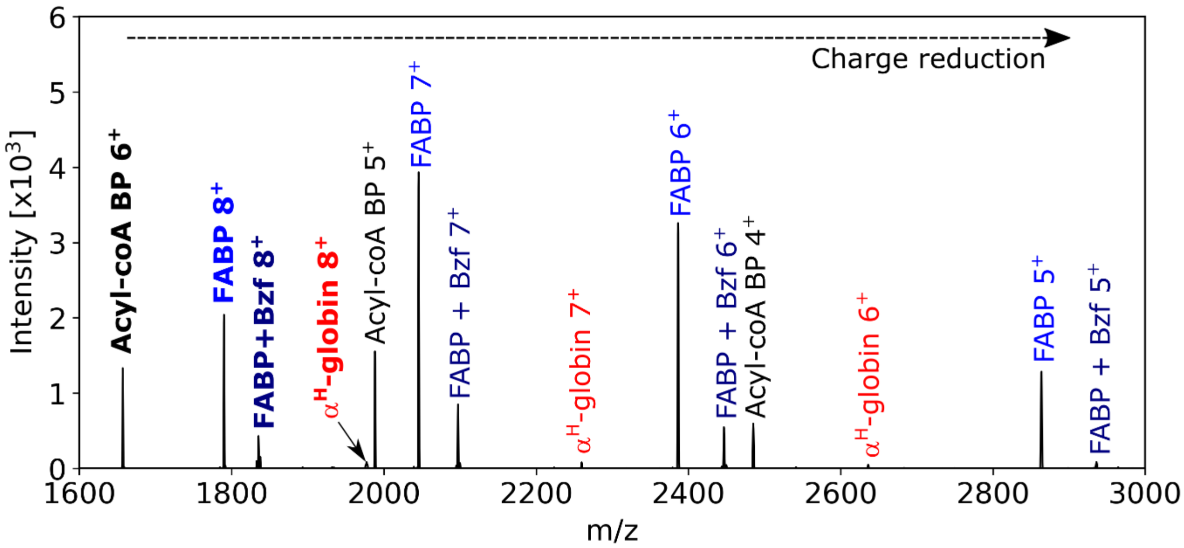

Figure S12: Multiplexed nano-DESI tPTCR mass spectrum (liver tissue, Animal 3) featuring signals monitored during MSI experiments. Precursor ion signals are labelled in bold. Precursor ions were isolated in series using the quadrupole mass filter and stored together in the IRM prior to simultaneous PTCR and *m/z* analysis at an orbitrap resolution = 7500 (*m/z* 200). Mass spectrum is the average of 163 scans within one nano-DESI line scan.

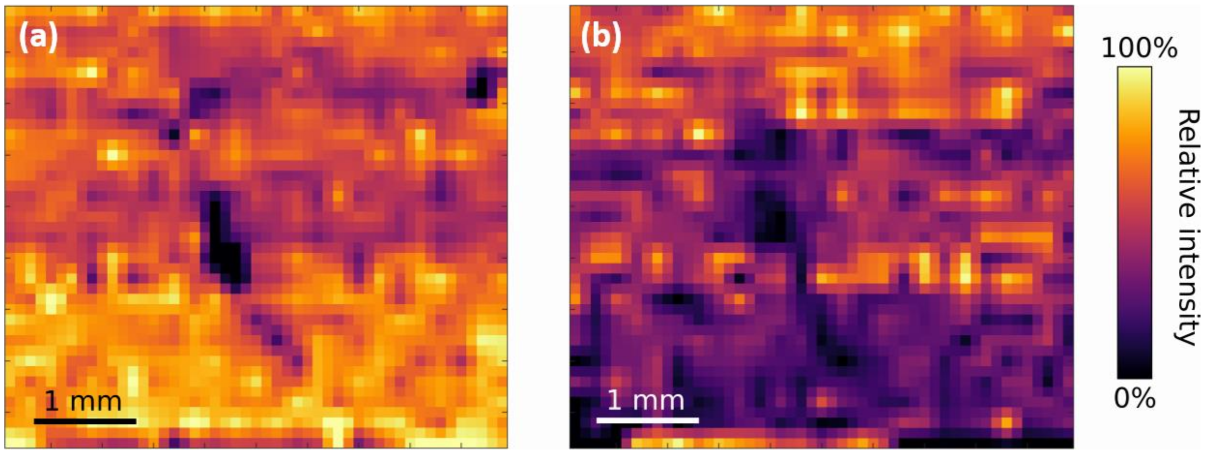

Figure S13: Targeted proton transfer charge reduction (tPTCR) nano-DESI mass spectrometry imaging of liver tissue from orally-dosed rat (Animal 3). Ion imagea composed from a) charge-reduced unbound FABP1 (7+ and 6+ charge states), and b) ACBP (5+ and 4+ charge states).

References

1. Randall, E. C.; Bunch, J.; Cooper, H. J., Direct analysis of intact proteins from *Escherichia coli* colonies by liquid extraction surface analysis mass spectrometry. *Anal. Chem.* **2014,** *86* (21), 10504-10510.
2. Hale, O. J.; Cooper, H. J., Native mass spectrometry imaging of proteins and protein complexes by nano-DESI. *Anal. Chem.* **2021,** *93* (10), 4619-4627.
3. D. LeDuc, R.; L. Kelleher, N., Using ProSight PTM and Related Tools for Targeted Protein Identification and Characterization with High Mass Accuracy Tandem MS Data. *Current Protocols in Bioinformatics* **2007,** *19* (1), 13.6.1-13.6.28.
4. https://prospector.ucsf.edu/prospector/mshome.htm. *UCSF Mass Spectrometry Facility and Pharmaceutical Chemistry*.
5. Robichaud, G.; Garrard, K. P.; Barry, J. A.; Muddiman, D. C., MSiReader: An open-source interface to view and analyze high resolving power MS imaging files on matlab platform. *J. Am. Soc. Mass Spectrom.* **2013,** *24*, 718-721.
6. Fellers, R. T.; Greer, J. B.; Early, B. P.; Yu, X.; LeDuc, R. D.; Kelleher, N. L.; Thomas, P. M., ProSight Lite: graphical software to analyze top-down mass spectrometry data. *Proteomics* **2015,** *15* (7), 1235-1238.
7. Hüttemann, M.; Pecina, P.; Rainbolt, M.; Sanderson, T. H.; Kagan, V. E.; Samavati, L.; Doan, J. W.; Lee, I., The multiple functions of cytochrome c and their regulation in life and death decisions of the mammalian cell: From respiration to apoptosis. *Mitochondrion* **2011,** *11* (3), 369-381.
8. Yergey, J. A., A general approach to calculating isotopic distributions for mass spectrometry. *International Journal of Mass Spectrometry and Ion Physics* **1983,** *52* (2), 337-349.
